# Development of an Oxidative Phosphorylation-Related and Immune Microenvironment Prognostic Signature in Uterine Corpus Endometrial Carcinoma

**DOI:** 10.3389/fcell.2021.753004

**Published:** 2021-11-25

**Authors:** Jinhui Liu, Tian Chen, Min Yang, Zihang Zhong, Senmiao Ni, Sheng Yang, Fang Shao, Lixin Cai, Jianling Bai, Hao Yu

**Affiliations:** ^1^ Department of Gynecology, The First Affiliated Hospital of Nanjing Medical University, Nanjing, China; ^2^ Department of Biostatistics, School of Public Heath, Nanjing Medical University, Nanjing, China

**Keywords:** uterine corpus endometrial carcinoma, oxidative phosphorylation, prognosis, tumor microenvironment, immunotherapy

## Abstract

**Background:** As the fourth most common malignant tumors in women, uterine corpus endometrial carcinoma (UCEC) requires novel and reliable biomarkers for prognosis prediction to improve the overall survival. Oxidative phosphorylation (OXPHOS) is found to be strongly correlated with the progression of tumor. Here, we aimed to construct an OXPHOS-related and immune microenvironment prognostic signature to stratify UCEC patients for optimization of treatment strategies.

**Method:** Prognosis-associated OXPHOS-related differentially expressed genes were identified by multivariable Cox regression from TCGA–UCEC cohort. Based on the candidate genes, an OXPHOS-related prognostic signature was constructed by the train set data and verified by the entire set. When integrated with relevant clinical characteristics, a nomogram was also created for clinical application. Through comparison of tumor microenvironment between different risk groups, the underlying mechanism of the model and the inner correlation between immune microenvironment and energy metabolism were further investigated.

**Results:** An OXPHOS-related signature containing ATP5IF1, COX6B1, FOXP3, and NDUFB11 was constructed and had better predictive ability compared with other recently published signatures in UCEC. Patients with lower risk score showed higher immune cell infiltration, higher ESTIMATE score (*p* = 2.808E−18), lower tumor purity (*p* = 2.808E−18), higher immunophenoscores (IPSs) (*p* < 0.05), lower expression of mismatch repair (MMR) proteins (*p* < 0.05), higher microsatellite instability (MSI), lower expression of markers of N6-methyladenosine (m6A) mRNA methylation regulators, higher tumor mutation burden (TMB) (*p* = 1.278E−9), and more sensitivity to immune checkpoint blockade (ICB) (*p* < 0.001) and chemotherapy drugs, thus, possessing improved prognosis.

**Conclusion:** An OXPHOS-related and immune microenvironment prognostic signature classifying EC patients into different risk subsets was constructed in our study, which could be used to predict the prognosis of patients and help to select a specific subset of patients who might benefit from immunotherapy and chemotherapy, thus, improving the overall survival rate of UCEC. These findings may contribute to the discovery of novel and robust biomarkers or target therapy in UCEC and give new insights into the molecular mechanism of tumorigenesis and progression of UCEC.

## 1 Introduction

Uterine corpus endometrial carcinoma (UCEC) is the fourth most common malignancy among women in the United States, with a trend of increasing morbidity and mortality worldwide (Cancer Genome Atlas Research et al., 2013). According to the GLOBOCAN, there would be an estimated 382,069 (2.1% of the total cancer cases) new cases, with 89,929 (0.9% of total cancer deaths) deaths in 2018 (Bray et al., 2018). Due to the advance in techniques for early diagnosis, the patients diagnosed at an early stage account for approximately 75% of UCEC, and most of the patients in stage I can be nearly cured by surgery, with a 5-years overall survival rate of 47–69%. In comparison, advanced-stage patients (stage III or IV) have a poor prognosis due to high risk for recurrence and limited therapeutic strategies, with a 5-years overall survival rate of 15–17% (Lee et al., 2017). Therefore, attention should be paid to discovering novel and reliable biomarkers for prognosis prediction, which can also work as a sensitive classifier for a specific subset of UCEC patients who will benefit from immunotherapy and have improved survival.

Recently, increasing attention has been paid into the exploration of the relationship between cancer cell metabolic plasticity and migration and metastasis (Mosier et al., 2021). Cancer cells with a more invasive and distal metastasis phenotype are found to be strongly correlated with the upregulated expression of peroxisome proliferator-associated receptor gamma and coactivator 1-alpha, which are hallmarks of active mitochondrial biogenesis and oxidative phosphorylation (OXPHOS) (LeBleu et al., 2014). OXPHOS is supposed to be a potential biomarker of tumor progression (Commander et al., 2020). In addition, emerging evidence shows that OXPHOS inhibition can be a valuable target in cancer treatment (Ashton et al., 2018; Molina et al., 2018; Xu et al., 2020a; Cardenas et al., 2020). OXPHOS provides the required energy for cancer cells to strive, and cancer stem cells with primary or acquired resistance against chemotherapy or tyrosine kinase inhibitors are characterized with markedly enhanced OXPHOS dependency (Sica et al., 2020). Metformin and thiazolidinediones can inhibit the mitochondrial electron transport chain, which provides energy for ovarian cancer growth. Thus, interfering with the process of OXPHOS may be considered as a new target for cancer therapy (Nayak et al., 2018). With the development of molecular medicine and next-generation sequencing technology, OXPHOS has been employed in the cancer risk prediction model in lung adenocarcinoma (Xu et al., 2020b). OXPHOS has also attached great importance to UCEC. As a regulator participating in oxidative phosphorylation and glycolysis, PKM2 can function as a biomarker for malignant and premalignant endometrial lesions, and the presence of PKM2^high^ tumor cells in UCEC tissue also indicates a poor prognosis (Lai et al., 2019). To the best of our knowledge, OXPHOS has not been included in the UCEC prognostic model in previous studies, which deserves to be explored for subgrouping of the patients with distinct prognosis for personal treatment and identifying potential novel therapeutic targets.

**FIGURE 1:**
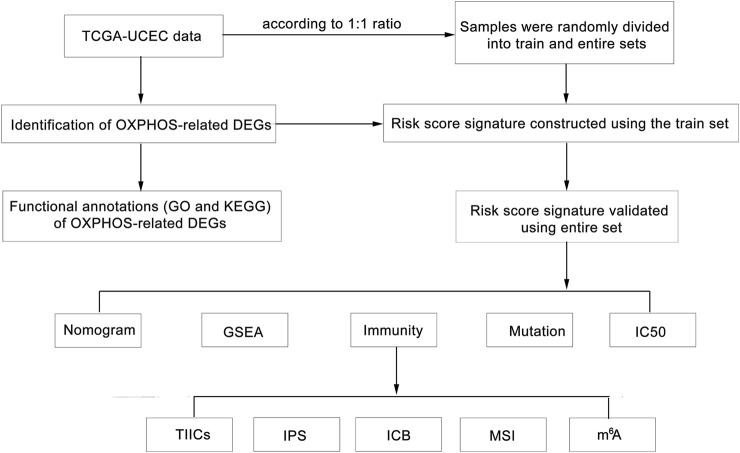
Flow chart of the bioinformatic analyses.

Here, we identified prognosis-associated OXPHOS-related differentially expressed genes (DEGs) from TCGA–UCEC cohort (Tomczak et al., 2015). Based on the candidate genes, we constructed an OXPHOS-related prognostic signature model using the train dataset and verified it using the entire dataset. To further investigate the underlying mechanism of the model and its correlation with immune microenvironment and energy metabolism, then between high- and low-risk groups, we compared tumor-infiltrating immune cells (TIICs), ESTIMATE score, tumor purity, immunophenoscores (IPSs), the expression of mismatch repair (MMR) proteins, mRNA expression-based stemness index (mRNAsi), microsatellite instability expression (MSI), the expression of markers of N^6^-methyladenosine (m^6^A) mRNA methylation regulators, tumor mutation burden (TMB) and the response to immune checkpoint blockade (ICB) and chemotherapy agents, which may provide evidence for clinical application of the signature for immunotherapy and chemotherapy option (Figure 1). The findings in this study not only provided a useful predictive model for UCEC but also offered evidence for mutual impacts of OXPHOS, immune microenvironment, and tumor malignant biology.

## 2 Materials and Methods

### 2.1 Data Collection

The gene expression profiles were collected from TCGA–UCEC database (https://tcga-data.nci.nih.gov/tcga/), which contained 552 tumor and 23 normal tissues. Meanwhile, the corresponding clinical and pathological information was also obtained from TCGA database, including patient age, tumor grade, tumor stage, and histological type of each sample. After removing the samples with unknown survival time, <30 days, and no survival status, we finally integrated the transcriptome and complete clinical data, enrolling a total of 511 patients in this study (Table 1).

**TABLE 1:** Clinical information of the included UCEC patients in TCGA.

Covariates	Type	Total N (Percentage)	Train N (Percentage)
Age	<=60	199 (38.94%)	109 (42.58%)
	>60	312 (61.06%)	147 (57.42%)
Histological type	Endometrial	384 (75.15%)	197 (76.95%)
	Mixed and serous	127 (24.85%)	59 (23.05%)
Grade	G1 and G2	91 (17.81%)	44 (17.19%)
	G3 and G4	420 (82.19%)	212 (82.81%)
Stage	Stage I & Stage II	370 (72.41%)	182 (71.09%)
	Stage III Stage IV	141 (27.59%)	74 (28.91%)

### 2.2 Oxidative Phosphorylation-Related Survival-Related Differentially Expressed Genes and Functional Annotation

We used the R package *limma* to define the differentially expressed genes among 289 OXPHOS-related genes acquired from the National Center for Biotechnology Information—GENE and the Metabolic Atlas database (Xu et al., 2020b). The genes with *p* < 0.05 and |log2 (fold change)| >1 were regarded as DEGs. We used the *survival* package to show the association between OXPHOS-related DEGs and survival time. We defined the DEGs with *p* less than 0.05 as survival-related DEGs (sDEGs). To explore the potential biological functions of the OXPHOS-related sDEGs, we implemented the *ClusterProfiler* package of R software for Gene Ontology (GO) term enrichment analysis (Ashburner et al., 2000), which included three categories: biological process (BP), molecular functions (MF), cellular components (CC), as well as the Kyoto Encyclopedia of Genes and Genomes (KEGG) pathway enrichment analysis (Kanehisa and Goto, 2000).

### 2.3 Construction of an Oxidative Phosphorylation-Related Prognostic Signature

We randomly assigned 256 patients as the train set and took the entire set for validation. First, we utilized the univariate Cox regression in the train set to identify candidate sDEGs with a significant level of *p* < 0.05. Then, we performed the least absolute shrinkage and selection operator (LASSO) Cox regression analysis by the *glmnet* package (Qu et al., 2018). Finally, we employed multivariate Cox proportional hazard regression analysis to construct an OXPHOS-related prognostic signature. The formula for calculating risk score was as follows: $\mathrm{risk}\;\mathrm{score}\;=\;\sum_{\mathrm{i=1}}^{n}\mathrm{the}\;\mathrm{expression}\;\mathrm{level}\;\mathrm{of}\;\mathrm{n*}$ the regression coefficient calculated by multivariate Cox proportional hazard regression.

We treated the median of risk score in the train set as the cutoff value to divide the validation data into the high- and low-risk groups and plotted the Kaplan–Meier curves, as well as the ROC curves by *survminer*, *survivalROC* package (Heagerty et al., 2000). To confirm the independence of the signature, a conjoint univariate and multivariate Cox analyses were conducted. In addition, we compared the subgroup survival analysis in tumor stage (stages I and II, stages III and IV), tumor grade (grades 1 and 2, grades 3 and 4), histological type (endometrial, mixed, and serous), and age (≤60, >60) to further evaluate the value of the signature combined with clinical factors in the survival prediction of UCEC. We used the *rms* package to construct a baseline nomogram for clinical application in UCEC patients (Iasonos et al., 2008).

### 2.4 RNA Isolation and Quantitative Real-Time PCR

UCEC and normal endometrial tissues were obtained from patients of the First Affiliated Hospital of Nanjing Medical University with the approval of the ethics committee and with informed consent. The clinicopathological parameters are shown in Supplementary Table S1. Total RNA was isolated from tissue samples with TRIzol reagent (Thermo Fisher Scientific, Waltham, MA, United States), and the integrity of the extracted RNA was estimated by the Agilent Bioanalyzer 2100 (Agilent Technologies, Santa Clara, CA, United States). RNA was reversely transcribed into cDNA using high-capacity reverse transcription kits (TaKaRa, Shiga, Japan), and quantitative real-time PCR (qRT-PCR) was conducted on Light Cycler 480 (Roche, Switzerland) using SYBR Green PCR Kit (Thermo Fisher Scientific) with the 2^−ΔΔCt^ method, in which GAPDH was the endogenous control. All program procedures of qRT-PCR were performed according to the protocol of the manufacturer. Primer sequences for GAPDH and four OXPHOS-related genes are presented in Supplementary Table S2.

### 2.5 Gene Set Enrichment Analysis and Immunity Analyses

Gene set enrichment analysis (GSEA) was utilized to elucidate the molecular mechanisms of the OXPHOS-related sDEGs. We separated the samples in the entire set into the high- and low-risk groups based on scores calculated by the signature and then compared the enriched BP between the two groups. The value of *p* < 0.05 was regarded as the cutoff criterion (Zhu et al., 2016). Furthermore, we employed the ESTIMATE algorithm by the *ESTIMATE* package to calculate the stromal, immune, and estimate scores of each sample and evaluate the relationship between the OXPHOS-related prognostic signature and immunization (https://bioinformatics.mdanderson.org/public-software/estimate/) (Yoshihara et al., 2013).

### 2.6 Analysis of Tumor-Infiltrating Immune Cells

We employed CIBERSORT (https://cibersort.stanford.edu/) to explore the immune cell infiltration of each sample based on the RNA-seq data (Newman et al., 2015). To further analyze the association between risk score and tumor-infiltrating immune cells (TIICs), single-sample gene-set enrichment analysis (ssGSEA) was also performed to quantify the immune activity in different risk groups by exploring 29 immune-related genes (He et al., 2018).

### 2.7 Analysis of Immune Status Between Different Risk Groups

The immunogenicity of tumor is determined by the four dominant components containing immunomodulators, immunosuppressive cells, effector cells as well as MHC molecules. Immunophenoscores (IPSs) (including IPS, IPS-CTLA4, IPS-PD1-PD-L1-PD-L2, IPS-PD1-PD-L1-PD-L2-CTLA4 scores) are calculated based on the representative cell-type gene expression z-scores to evaluate and compare the potential response to immune checkpoint inhibitor between different risk groups (Charoentong et al., 2017). IPS data of each UCEC patient in the entire set was downloaded from The Cancer Immunome Atlas (TCIA) (https://tcia.at/home). Besides, the immunotherapy response could be predicted with high accuracy by an Immune Cell Abundance Identifier (ImmuCellAI) result-based model, which precisely estimates the abundance of 24 immune cell types including 18 T-cell subsets from gene expression data (http://bioinfo.life.hust.edu.cn/ImmuCellAI) (Wang et al., 2020a; Toh et al., 2021). Microsatellite instability (MSI) (Toh et al., 2021) and the expression of markers of N^6^-methyladenosine (m^6^A) mRNA methylation regulators (Wang et al., 2020a) of each sample were also collected from TCGA, which can predict potential response to immune checkpoint inhibitors (ICIs) and help explain the association between the signature and immunogenicity. Tumor cells sharing more similarities with stem cells represents cancer progression. Malta et al. developed an innovative mRNAsi algorithm to calculate the degree of similarities for UCEC patients in TCGA database (Malta et al., 2018). We also downloaded the mRNAsi information from the research for analyses.

### 2.8 Analysis of Tumor Mutation Burden

The mutation data collected from TCGA were analyzed by R package maftools (Robinson et al., 2017). The tumor mutation burden (TMB) was calculated by the formula: TMB = (total mutation/total covered bases) * $10^{6}$ .

### 2.9 Potential Chemotherapeutic Response

The response to temsirolimus, roscovitine, AZD6244, PD.0325901, RDEA119, PF.02341066, AKT. inhibitor.VIII, BMS.509744, vinblastine, bryostatin.1, metformin, AZ628, nutlin.3a, bortezomib, bicalutamide, AZD6482, cytarabine, rapamycin, camptothecin, mitomycin. C, S. Trityl.L.cysteine, tipifarnib, parthenolide, sorafenib, and methotrexate, the 25 common chemo drugs, were predicted by the Genomics of Drug Sensitivity in Cancer (GDSC) (https://www.cancerrxgene.org/) to analyze the relationship between the signature and chemotherapeutic response (Yang et al., 2013). We used R package *pRRophetic* to estimate and compare the half-maximal inhibitory concentration (IC50) between different risk groups (Geeleher et al., 2014).

### 2.10 Statistical Analysis

All statistical analyses were applied by R version 3.6.1 (Package: *limma*, *survival*, *ClusterProfiler*, *glmnet*, *survminer*, *survivalROC*, *rms*, *ESTIMATE, pRRophetic*). Categorical variables were presented by counts and percentages. Continuous variables in normal distribution were analyzed using Student’s t-test and presented as mean ± standard deviation, while in abnormal distribution, they were presented as median (range). Multiple groups of continuous variables were analyzed by one-way ANOVA. The hazard ratio and 95% confidence interval were calculated to identify genes associated with overall survival. Unless with special explanation, *p* < 0.05 was considered statistically significant.

## 3 Results and Discussion

### 3.1 Results

#### 3.1.1 Identification of Oxidative Phosphorylation-Related Differentially Expressed Genes

We conducted the differential expression analysis of 289 OXPHOS-related genes between tumor and normal endometrium (Xu et al., 2020b). Sixty-six OXPHOS-related DEGs were identified, including 54 upregulated genes and 12 downregulated genes (Supplementary Figures S1A,B and Supplementary Table S3). The GO term enrichment analysis presented that they were related to ATP metabolic process (*p* < 0.0005) and purine ribonucleoside triphosphate metabolic process (*p* < 0.0005) in the BP group, mitochondrial inner membrane (*p* < 0.0005), and organelle inner membrane (*p* < 0.0005) in the CC group, oxidoreductase activity, acting on NAD(P)H and electron transfer activity (*p* < 0.0005) in the MF group (Supplementary Figures S1C); the KEGG results manifested that they mainly took part in pathways of oxidative phosphorylation (*p* < 0.05), endocannabinoid signaling (*p* < 0.05), and HIF-1 signaling (*p* < 0.05) in cancer (Supplementary Figures S1D).

#### 3.1.2 Establishment of Oxidative Phosphorylation-Related Prognostic Signature

Using univariate Cox regression from the train data, we defined seven OXPHOS-related sDEGs, including ATP5IF1, MRPL12, FOXP3, NDUFA13, ATP5F1E, and NDUFB11 (Supplementary Table S4). ATP5IF1 and FOXP3 were protective elements of UCEC, while the others were dangerous ones. Then we conducted LASSO Cox regression analysis and multivariate Cox regression analysis (Supplementary Figures S2A,B) to construct a reliable OXPHOS-related prognostic signature. Consequently, ATP5IF1, COX6B1, FOXP3, and NDUFB11 were included in the signature as key prognosis-associated ones (Supplementary Figures S2C). Furthermore, the results of the expression analyses of the four genes showed that all of them were highly expressed in tumor compared with normal tissue, but only ATP5IF1 and FOXP3 were highly associated with overall survival (OS) of UCEC patients (Supplementary Figures S3A–F). The qRT-PCR results of our own samples also demonstrated that the expression level of ATP5IF1 (*p* = 0.0311), COX6B1 (*p* = 0.0212), FOXP3 (*p* = 0.1082), and NDUFB11 (*p* = 0.0450) were significantly higher in UCEC tissues than in normal endometrial tissues (Supplementary Figures S3G–J). The formula to calculate the risk score of patients is shown as follows: risk score = (−0.011678 * expression value of ATP5IF1) + (0.0014649 * expression value of COX6B1) + (−0.309569 * expression value of FOXP3) + (0.0029791 * expression value of NDUFB11).

#### 3.1.3 Validation of Prognostic Signature in Uterine Corpus Endometrial Carcinoma

**FIGURE 2:**
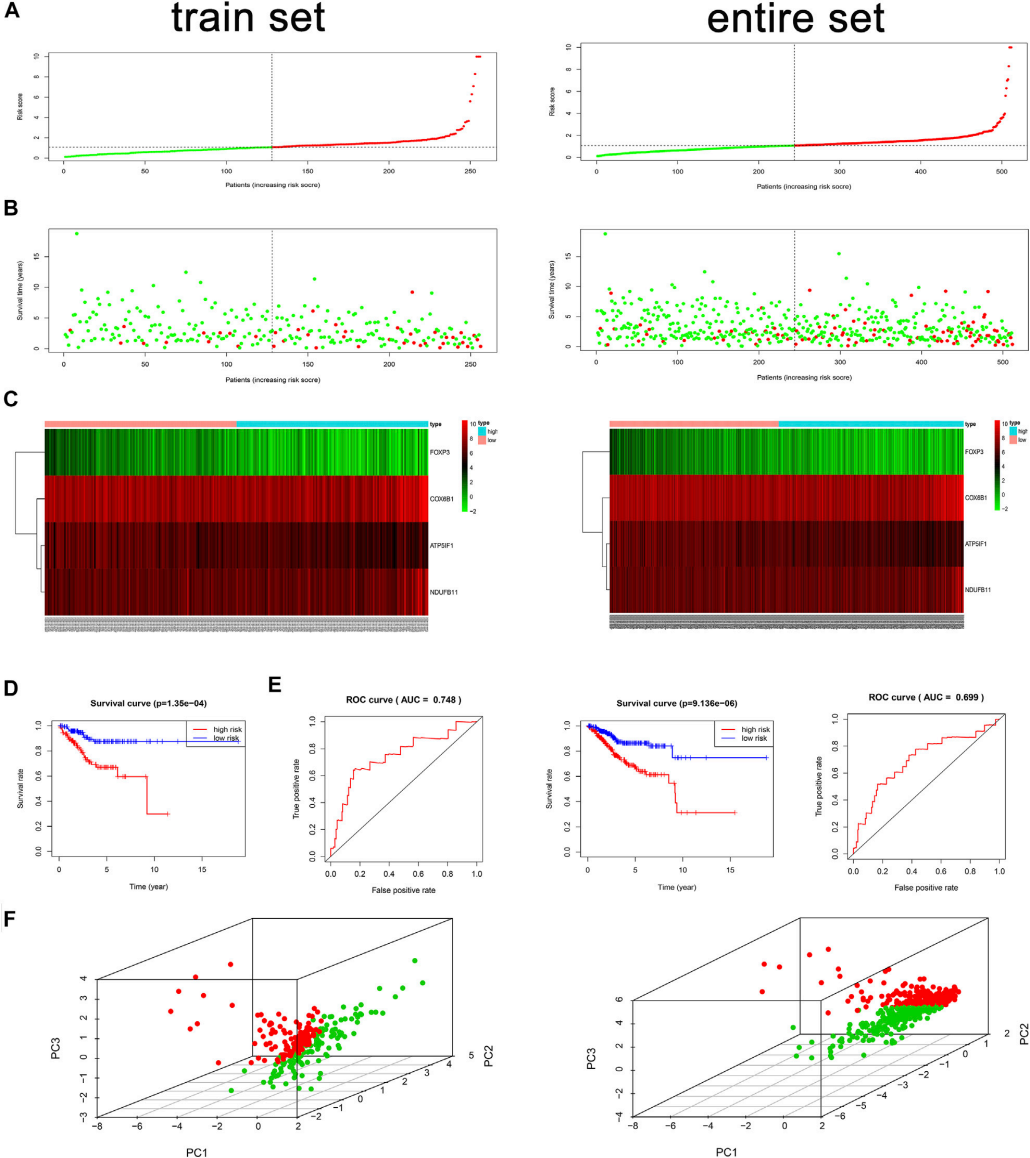
Risk score analysis, time-dependent ROC analysis, Kaplan–Meier analysis, and PCA plots for the validation of the oxidative phosphorylation (OXPHOS)-related prognostic signature in the train and entire sets based on overall survival (OS). **(A)** Rank of risk score and distribution of groups based on the signature. Patients in each group were divided into the high- or low-risk groups. **(B)** Survival status of patients in each group. **(C)** Heatmap of expression profiles of selected four OXPHOS-related prognostic differentially expressed genes (DEGs). **(D)** Kaplan–Meier survival curves analyses of risk score in TCGA cohort. In the train set, in the low-risk group, *N* = 128; in the high-risk group, *N* = 128. In the entire set, in the low-risk group, *N* = 244; in the high-risk group, *N* = 267. **(E)** Survival ROC analysis of risk score in predicting prognoses in the train and entire sets. **(F)** PCA plots of the train and entire sets. In the train set, *N* = 256; in the entire set, *N* = 511.

We used the validation data to evaluate the performance of the risk model in survival prediction. Using the preceding equation, we calculated the risk score and divided the samples of the train and entire sets separately into high- and low-risk groups by taking the median risk score in the train set as the cutoff line. The distribution of risk score (Figure 2A), survival status of each sample (Figure 2B), and the key gene expression profiles in each set (Figure 2C) are exhibited in Figure 2. By comparison with the low-risk group, Kaplan–Meier survival curves revealed that the OS of the high-risk group was significantly worse (log rank test, *p* = 1.35E−4) (Figure 2D). Besides, a survival ROC curve analysis was conducted, and the area under the curve (AUC) at 1 year in the train set was 0.748 (Figure 2E), which verified the predictive value of the signature, and the AUC value in the entire set also suggested a similar potential for prediction of survival with the above results (Figure 2E). The PCA plots indicated that patients in different risk groups tended to distribute differently (Figure 2F).

#### 3.1.4 Clinical Utility of the Oxidative Phosphorylation-Related Prognostic Signature

**FIGURE 3:**
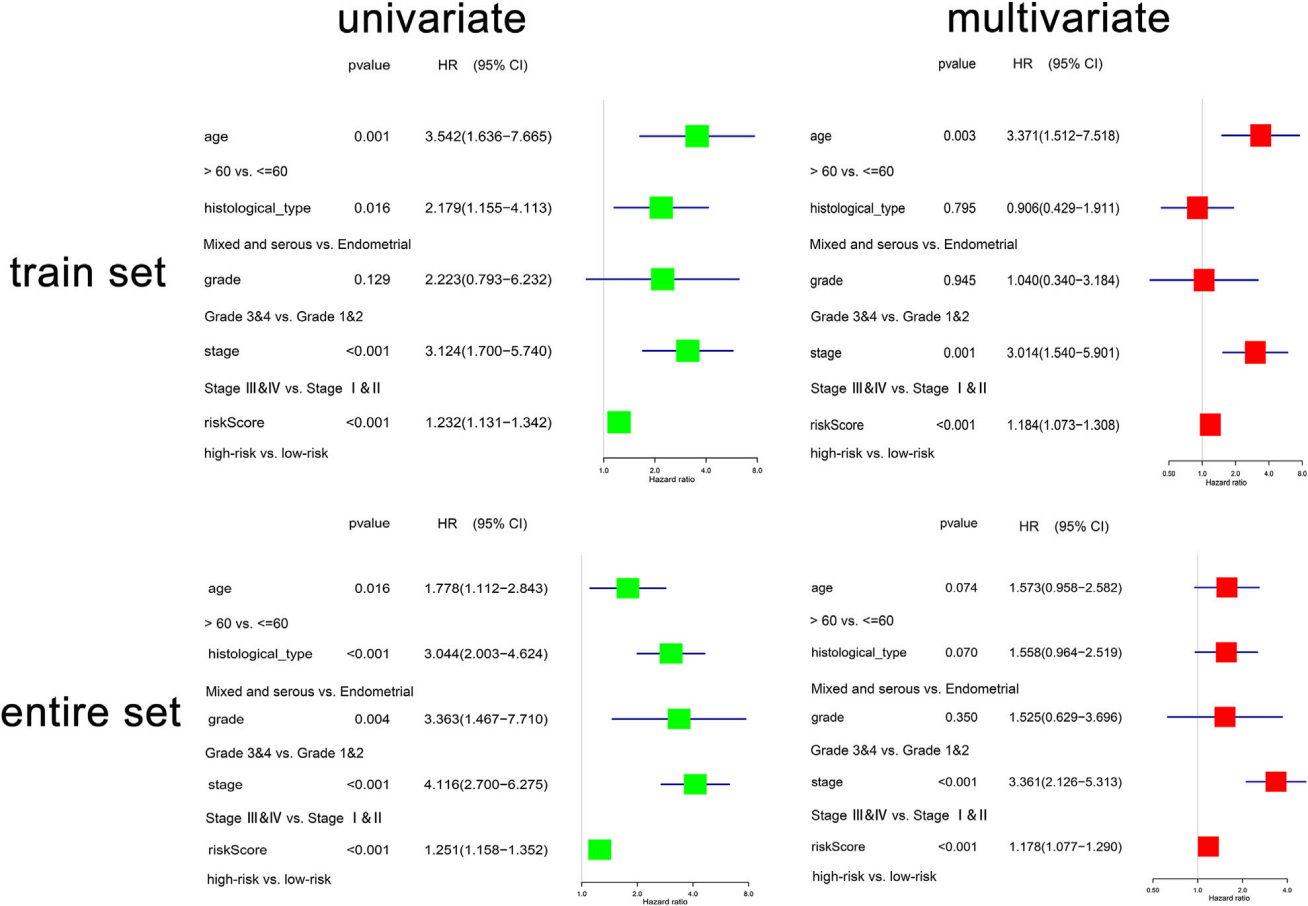
The regression analyses of the independent predictor of uterine corpus endometrial carcinoma (UCEC). In the train set, *N* = 256; in the entire set, *N* = 511.

**FIGURE 4:**
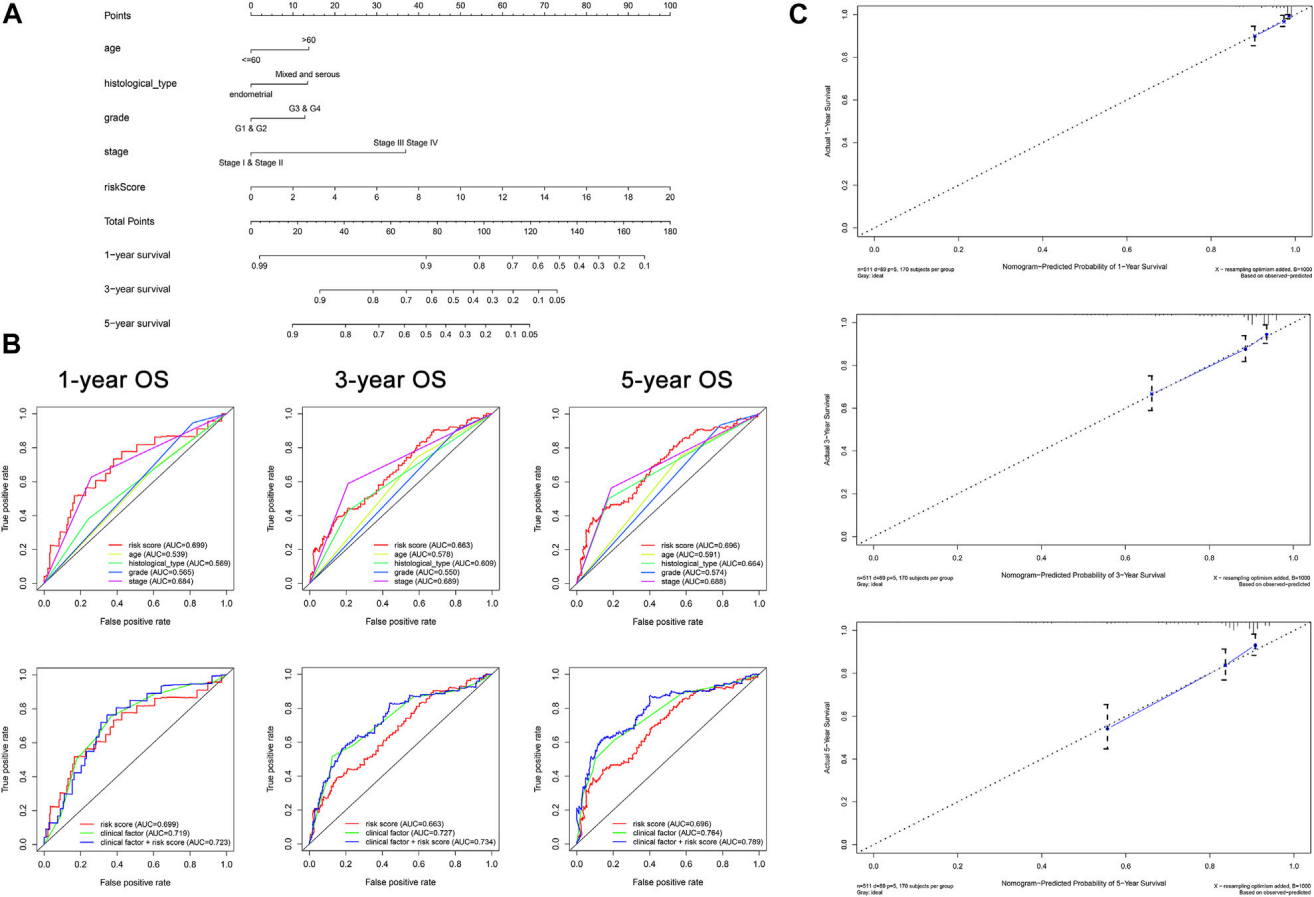
Construction of nomogram for predicting survival of patients with UCEC (*N* = 511). **(A)** The baseline nomogram including five factors: patient age, histological type, tumor grade, tumor stage, and risk score. **(B)** ROC curves of 1-, 3-, and 5-years OS indicated that the gene-based signature had better predictive ability compared with other clinical characteristics. In addition, the nomogram composed of the signature and clinical characteristics showed superior prediction value for the prognosis. **(C)** Calibration plots of 1-, 3-, and 5-years OS suggested that the prognosis predicted by the nomogram was consistent with the actual outcome.

The conjoint univariate and multivariate analyses in both sets confirmed the independence of the OXPHOS-related prognostic signature (Figure 3). Integrated with clinical factors, we performed subgroup survival analysis in stages I and II, stages III and IV, grades 1 and 2, grades 3 and 4, endometrial, mixed, and serous, age ≤6 0, age >60, and we found that a higher risk score was related to poorer prognosis in all subgroups (Supplementary Figures S4A–H). The correlation between risk core and clinical characteristics is shown in Supplementary Figures S4I–L and a heatmap (Supplementary Figure S5), indicating that patients with lower stage, lower grade, endometrial histological type, and lower age tended to have a lower risk score. Aimed at creating a feasible method for clinical application, we constructed a nomogram cooperating risk core, patient age, histological type, tumor grade, and tumor stage (Figure 4A). By comparing the AUC values at 1, 3, and 5 years plotted by the signature and clinical factors, we discovered that the gene-based signature was superior to others in the OS prediction. What is more, when the gene-based signature was combined with the clinical characteristics, it had better predictive ability than the signature alone (Figure 4B). The calibration curves also demonstrated that the results predicted by the nomogram were in high consistency with actual survival, which suggested that the nomogram had a high clinical value (Figure 4C).

#### 3.1.5 Gene Set Enrichment Analyses

**FIGURE 5:**
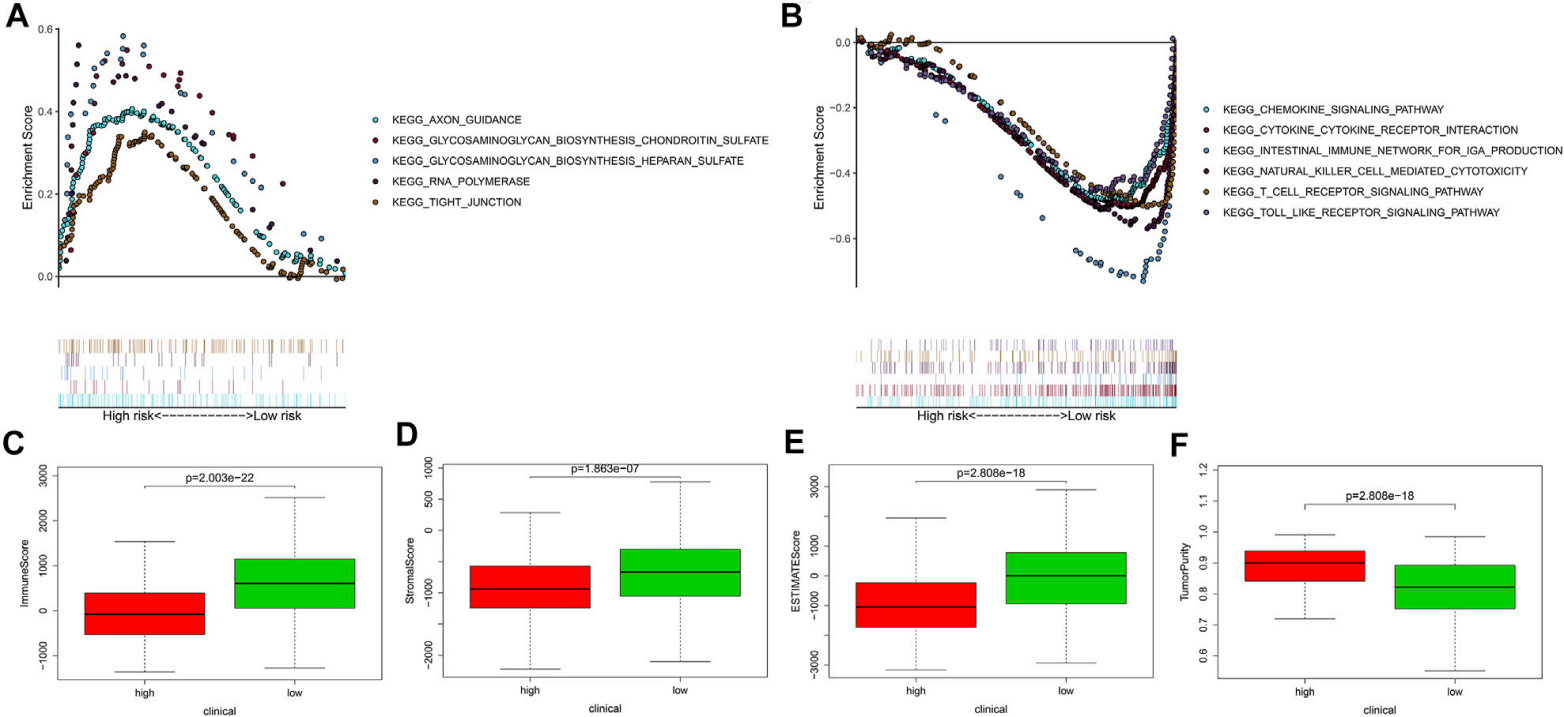
The gene set enrichment analysis, the immune, stromal, and ESTIMATE scores, and the tumor purity of different risk groups. **(A,B)** The gene set enrichment analysis showed the significantly enriched Kyoto Encyclopedia of Genes and Genomes (KEGG) pathways in TCGA. **(C–F)** The AVONA results of the immune, stromal, and ESTIMATE scores and tumor purity in the high- and low-risk groups. Patients in the high-risk group had lower immune score, stromal score, ESTIMATE score, and higher tumor purity. In the high-risk group, *N* = 267; in the low-risk group, *N* = 244.

To ascertain involved biological processes, we employed GSEA to analyze the transcript message of patients in different risk groups. In the high-risk group, representative KEGG pathways were axon guidance, glycosaminoglycan biosynthesis ghondroitin sulfate, glycosaminoglycan biosynthesis heparan sulfate, RNA polymerase, and tight junction (Figure 5A), while in the low-risk group, representative KEGG pathways were cytokine–cytokine receptor interaction, chemokine signaling pathway, and so on (Figure 5B). Through comparison, we discovered that in the low-risk group, the representative biological processes were mainly related to immunization. Furthermore, we implemented the ESTIMATE algorithm and found that the higher the risk score, the lower the immune score, the stromal score, and the ESTIMATE score, and the higher the tumor purity (Figures 5C–F), which further proved that the signature was significantly related to immunization.

#### 3.1.6 Difference of Tumor-Infiltrating Immune Cells in Different Risk Groups

**FIGURE 6:**
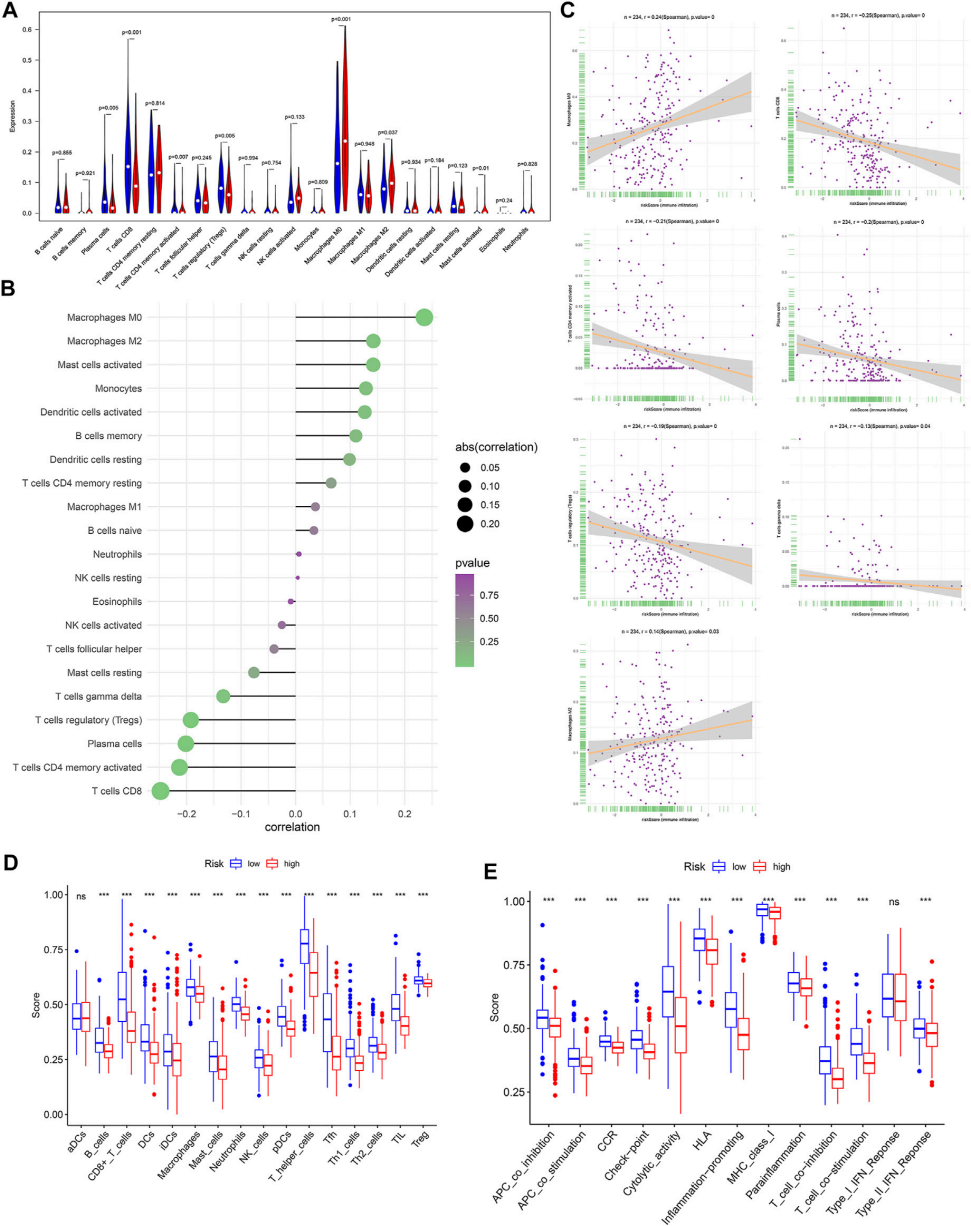
The relationship between risk score and the tumor-infiltrating immune cells (TIICs). **(A)** Comparison of the TIICs between different risk groups. **(B)** The correlation of risk score and TIICs. **(C)** The associations between M0 macrophages, M2 macrophages, plasma cells, activated memory CD4^+^ T cells, CD8^+^ T cells, gamma delta T cells, regulatory T cells (Tregs), and risk score. Comparison of the single-sample gene-set enrichment analysis (ssGSEA) scores between different risk groups in TCGA cohort, including the scores of 16 immune cells **(D)** and 13 immune-related functions **(E)** displayed in boxplots. CCR, cytokine–cytokine receptor. Adjusted *p*-values were shown as ns, not significant; *p < 0.05; **p < 0.01; ***p < 0.001. In the high-risk group, *N* = 267; in the low-risk group, *N* = 244.

To further explore the relationship between the OXPHOS-related prognostic signature and immunization, we found the difference of TIICs between different risk groups. M0 macrophages, M2 macrophages, and activated mast cells were significantly elevated in the high-risk group (*p* < 0.05). In contrast, plasma cells, CD8^+^ T cells, activated memory CD4^+^ T cells, and regulatory T cells (Tregs) were evidently elevated in the low-risk group (*p* < 0.05) (Figure 6A). We analyzed the correlation of risk score and TIICs; as we can see in Figures 6B,C, M0 macrophages, M2 macrophages, plasma cells, activated memory CD4^+^ T cells, CD8^+^ T cells, gamma delta T cells, and regulatory T cells (Tregs) were the seven most relevant types of immune cells with risk core. There was a significant difference in the enrichment scores of diverse immune cell subpopulations and related functions or pathways with ssGSEA between different risk groups, such as TIL and HLA, which showed coherence with the GO and KEGG analyses results (Figures 6D,E).

#### 3.1.7 Comparison of Immune Status Between Different Risk Groups

**FIGURE 7:**
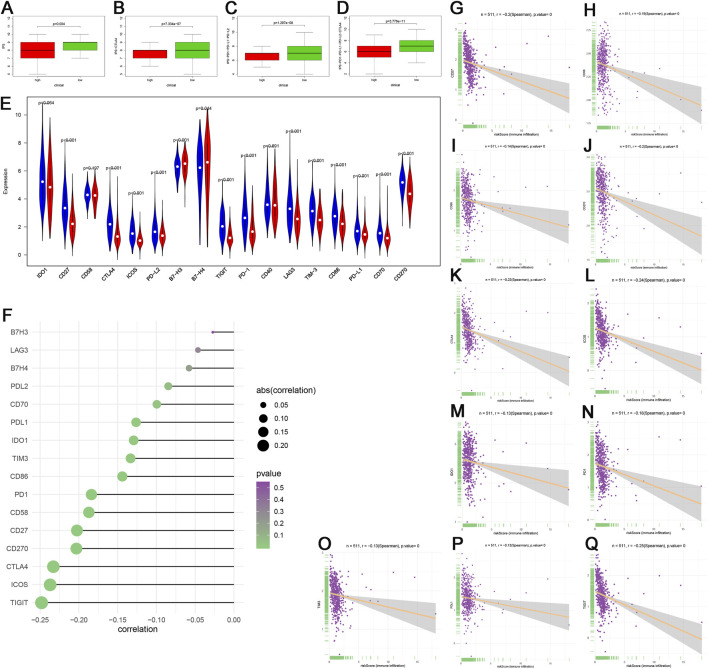
Immunophenoscore (IPS) and gene expression analyses of immune checkpoint. **(A–D)** Patients in the low-risk group had higher scores of IPS, IPS-PD1-PD-L1-PD-L2, IPS-CTLA4, and IPS-PD1-PD-L1-PD-L2-CTLA4 than patients in the high-risk group. **(E)** Comparison of the expression of immune checkpoint between the different risk groups. **(F–Q)** The associations among TIGIT, ICOS, CTLA4, CD27, CD270, CD58, PD-1, CD86, TIM-3, IDO1, PD-L1, and risk score. In the high-risk group, *N* = 267; in the low-risk group, *N* = 244.

IPS and the expression of immune modulators are able to predict the potential response of the patients to the immune checkpoint inhibitor. The scores of IPS (*p* = 0.004), IPS-PD1-PD-L1-PD-L2 (*p* = 1.297E−8), IPS-CTLA4 (7.334E−7), and IPS-PD1-PD-L1-PD-L2-CTLA4 (*p* = 3.776E−11) in the high-risk group were clearly higher than that in the low-risk group (Figures 7A–D). The expression of immune modulators including CD27, CTLA4, ICOS, PD-L2, TIGIT, PD-1, LAG3, TIM-3, CD86, PD-L1, CD70, and CD270 (*p* < 0.001) in the low-risk group was also remarkably higher than in the high-risk group (Figure 7E). We further discovered that the genes related to immune checkpoint inhibitor (ICI), including TIGIT, ICOS, CTLA4, CD27, CD58, PD-1, CD86, TIM-3, IDO1, and PD-L1 were negatively related to risk score (Figures 7F–Q), which suggested that the patients in the low-risk group promised to respond to immunotherapy.

**FIGURE 8:**
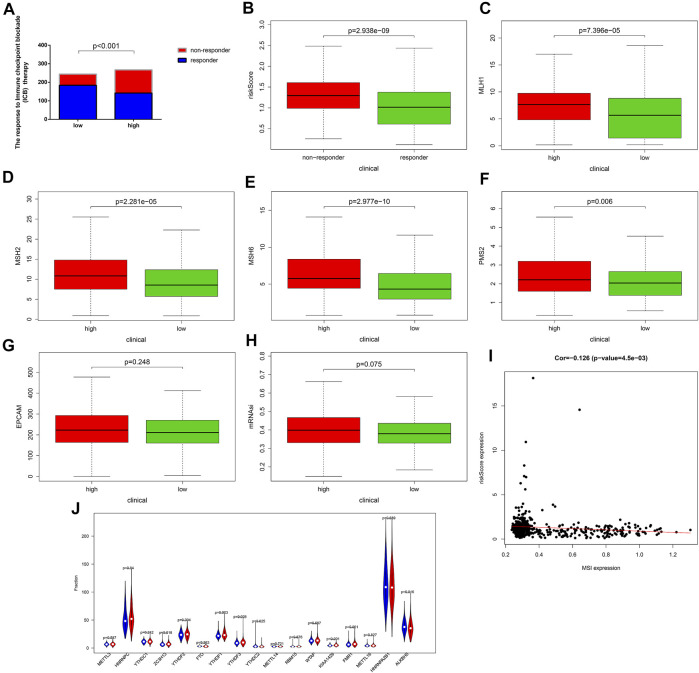
The response to immune checkpoint blockade (ICB) therapy and the microsatellite instability (MSI) status of different risk groups. **(A)** Comparison of the response to immune checkpoint blockade (ICB) therapy between the low- and the high-risk groups. Patients in the low-risk group showed more sensitive response to ICB therapy than those in the high-risk group. **(B–G)** Patients in the low-risk group had lower expression of the dominant mismatch repair (MMR) proteins including MLH1, MSH2, MSH6, PMS2, and EPCAM than patients in the high-risk group. **(H)** The mRNAsi of the low-risk group is lower than that of the high-risk group. **(I)** The correlation between the MSI expression and the risk score. **(J)** The expression of the markers of m^6^A mRNA methylation regulators in the high- and low-risk groups. m^6^A, N^6^-methyladenosine. In the high-risk group, *N* = 267; in the low-risk group, *N* = 244.

By comparing the reaction to immune checkpoint blockade (ICB), we found that the percentage of patients with response to ICB in the high-risk group (53.18%, 142/267) was lower than that in the low-risk group (75.41%, 184/244) (Figure 8A). The risk scores of the responders were also lower than those of the nonresponders (Figure 8B). These results could demonstrate that the OXPHOS-related prognostic signature had the potential to predict the possible effect of ICB in UCEC patients and work as a classifier for therapy selection.

Moreover, we compared the MSI status of different risk groups and found, as shown in Figures 8C–G, the expression level of dominant mismatch repair (MMR) proteins, such as MLH1, MSH2, MSH6, PMS2, and EPCAM, in the low-risk group were lower, and risk score was negatively correlated with the MSI expression (Figure 8I). The patients in the low-risk group had a lower score of mRNAsi (Figure 8H), which can partly explain the poor prognosis in the high-risk group. In addition, significantly decreased gene expression of KIAA1429, FMR1, HINRNPC, ZC3H13, YTHDF1, and YTHDF3, which are key factors of m^6^A mRNA methylation could be found in Figure 8J. These findings all provided evidence for the efficiency of immunotherapy for patients in the low-risk group and helped to elucidate the mechanisms of different outcomes.

#### 3.1.8 Relationship Between Tumor Mutational Burden and Risk Score

**FIGURE 9:**
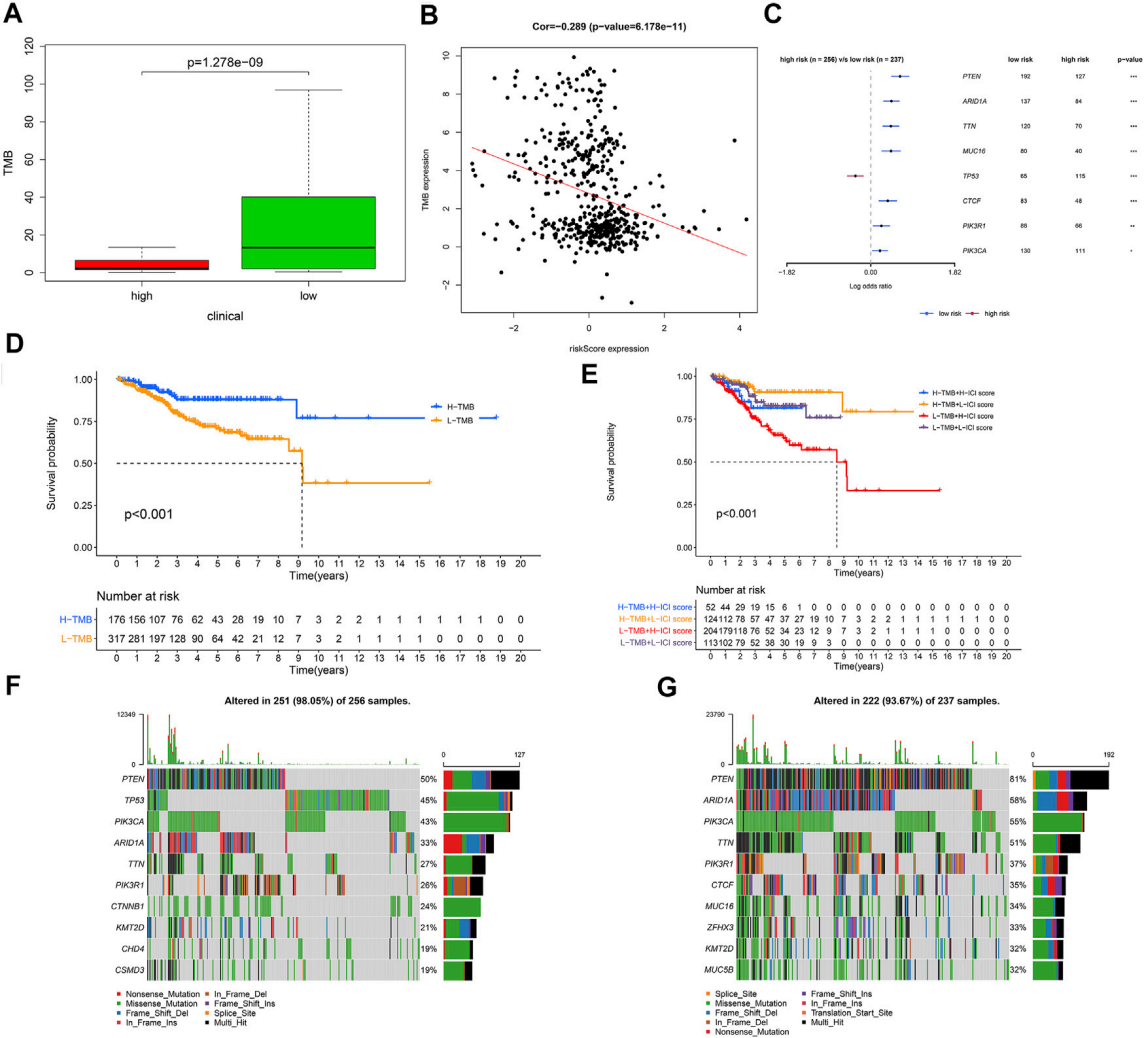
The relationship between risk score and tumor mutational burden (TMB). **(A)** Comparison of TMB between the low- and high-risk groups. **(B)** The correlation between risk score and TMB was negative. **(C)** The regression analyses of the mutated genes. **(D)** Kaplan–Meier curves for the high and low TMB groups of the TCGA–UCEC cohort. Log-rank test, *p* < 0.001. **(E)** Kaplan–Meier curves for patients in the TCGA–UCEC cohort stratified by both TMB and risk score. Log-rank test, *p* < 0.001. **(F)** Oncoplot displaying the somatic landscape of UCEC in the high-risk group. **(G)** Oncoplot displaying the somatic landscape of UCEC in the low-risk group. In the high-risk group, *N* = 267; in the low-risk group, *N* = 244.

To further clarify the mechanism of tumorigenesis and development of UCEC patients, we compared the tumor mutational burden (TMB) between different risk groups based on somatic mutation data. We discovered that the low-risk group has remarkably higher TMB than the high-risk group (*p* = 1.278E−8) (Figure 9A). The correlation between risk score and TMB was negative (Cor = −0.269, *p* = 6.178E−11) (Figure 9B). We also found that only the mutation of TP53 was found in the high-risk group, whereas the others were found in the low-risk group (Figure 9C). Next, we divided UCEC patients into H-TMB and L-TMB groups based on the median TMB; as shown in Figure 9D, the OS of the H-TMB group was better than that of the L-TMB group (log-rank test, *p* < 0.001). Stratified survival analysis was conducted to assess the synergistic effect of these factors, which corroborated that TMB would not influence the effect of the signature in prognosis prediction. Significant survival differences were observed in both TMB-stratified subgroups [log-rank test, H-TMB and high risk score (HH) versus H-TMB and low risk score (HL), *p* < 0.001; L-TMB and high risk score (LH) versus L-TMB and low risk score (LL), *p* < 0.001; Figure 9E], which indicated that the signature could function as an independent predictive indicator and effectively evaluate the potential response to immunotherapy. The most frequent somatic mutations in the high-risk group were as follows: PTEN > TP53 > PIK3CA > ARID1A > TTN > PIK3R1 > CTNNB1 > KMT2D > CHD4 > CSMD3 (Figure 9F). While the most frequent somatic mutations in the low-risk group followed the order PTEN > ARID1A > PIK3CA > TTN > PIK3R1 > CTCF > MUC16 > ZFHX3 > KMT2D > MUC5B (Figure 9G). Other mutation details, including variant classification, mutation type, SNV class, mutation load, and top 10 mutated genes in UCEC between different risk groups, are shown in Supplementary Figure S6.

#### 3.1.9 The Chemotherapeutic Response Between Different Risk Groups

**FIGURE 10:**
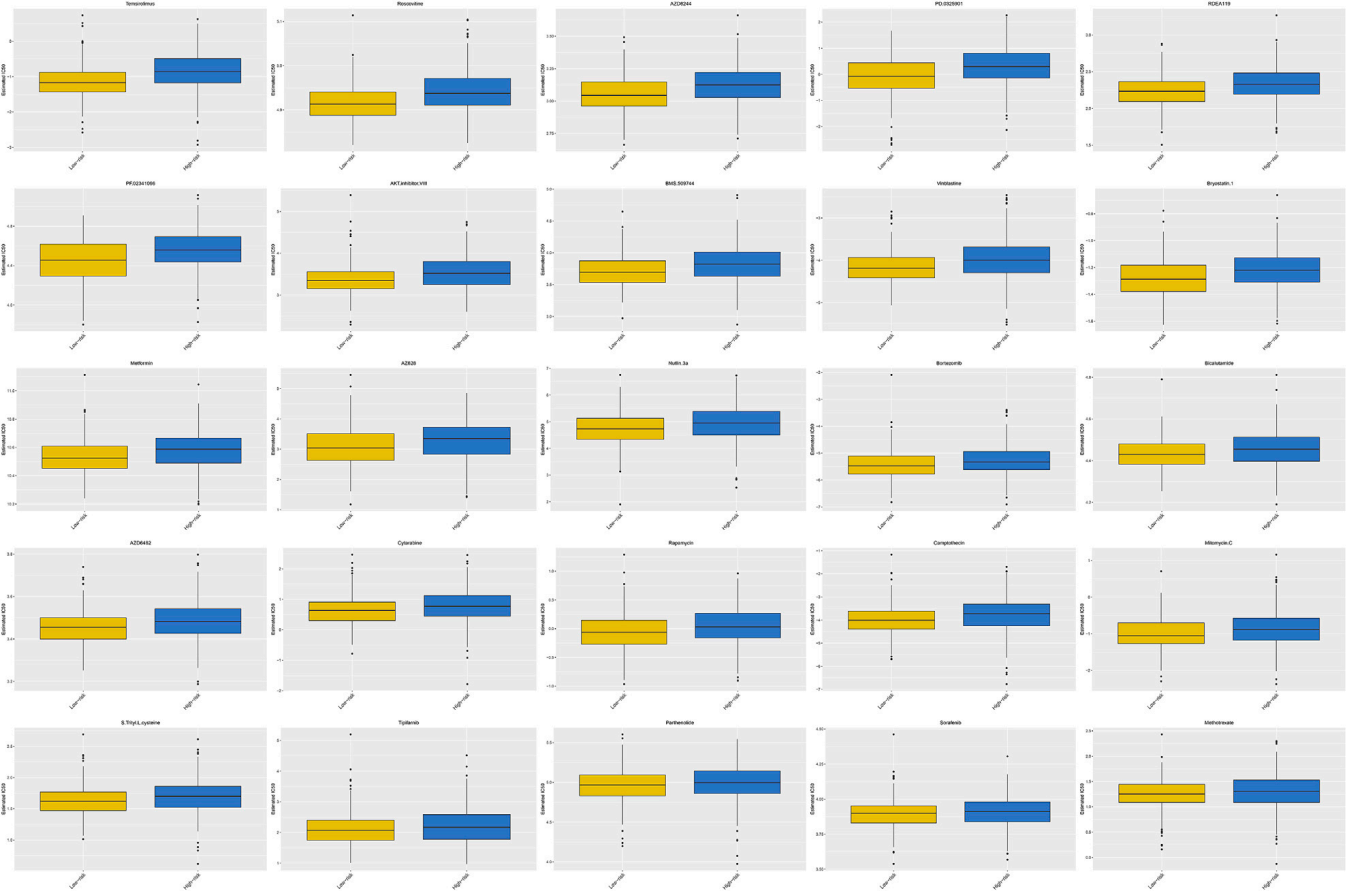
Prediction of the response to common chemotherapy drugs between the low- and high-risk groups. Patients in the high-risk group possessed higher estimated IC50 than patients in the low-risk group. In the high-risk group, *N* = 267; in the low-risk group, *N* = 244.

The high-risk group had a higher estimated IC50 of temsirolimus, roscovitine, AZD6244, PD.0325901, RDEA119, PF.02341066, AKT. inhibitor.VIII, BMS.509744, vinblastine, bryostatin.1, metformin, AZ628, nutlin.3a, bortezomib, bicalutamide, AZD6482, cytarabine, rapamycin, camptothecin, mitomycin. C, S. Trityl.L.cysteine, tipifarnib, parthenolide, sorafenib, and methotrexate than the low-risk group (*p* < 0.0001), which demonstrated that patients with a low risk score were more sensitive to these drugs (Figure 10). Therefore, the OXPHOS-related prognostic signature could also be utilized as a robust and valuable classifier for chemotherapy.

## 4 Discussion

Though the overall survival state of UCEC patients has improved a lot due to the development of early diagnostic techniques (Braun et al., 2016), there are still 15% of patients with UCEC diagnosed at an advanced stage, thus, suffering from tumor recurrence owing to a limited response to unsuitable therapies (Li and Wan, 2020). Therefore, developing valid and reliable biomarkers for survival prediction and treatment selection to avoid overtreatment in patients who will not relapse and propose individual adjuvant treatment to patients who will recur counts (Lee et al., 2017), and efforts have been put into developing a robust prognostic signature based on DEGs for identification of biologic subsets to guide treatment strategies for UCEC patients, such as a cell cycle-related prognostic signature (Liu et al., 2020a), an autophagy-related prognostic signature (Wang et al., 2020b), and so on. However, there is no consensus on predictive biomarkers for scientifically validated therapy.

In recent years, emerging evidence has proved that OXPHOS could function as a new target in cancer therapy (Ashton et al., 2018). However, OXPHOS has not been involved in developing a prognostic signature for UCEC, thus far, and the potential underlying mechanism and possible clinical application of OXPHOS-related signature in cancer are worth exploring.

Based on the 289 OXPHOS-related gene matrix obtained from the research of Zihao Xu, we used differential expression analysis and identified 66 OXPHOS-related DEGs, including 54 upregulated genes and 12 downregulated genes; most of them have been reported to be associated with cancer. For instance, EPAS1 could enhance the effect of paclitaxel on breast cancer cells by inhibiting growth and promoting apoptosis of MCF-7/TAX cells (Song et al., 2020). Furthermore, functional annotation of the OXPHOS-related DEGs also indicated that the candidate genes were significantly related to ATP metabolic process, purine ribonucleoside triphosphate metabolic process, and the essential pathways reported in many cancers (Matsuda et al., 2017; Fallah and Rini, 2019), including retrograde endocannabinoid signaling and HIF-1 signaling pathways.

Then, we randomly selected samples from the entire set in half into the train set and constructed the model based on it. After univariate Cox analysis and LASSO Cox regression analysis, finally, a prognostic model composed of four OXPHOS-related genes (ATP5IF1, COX6B1, FOXP3, and NDUFB11) was built. Except for ATP5IF1, other candidate genes included in the prognostic signature have been proven to play a vital role in cancer development. COX6B1, as one of the core proteins involved in the molecular basis of the below-background radiation stress response, might inhibit the proliferation of laryngeal squamous cell carcinoma cells (Liu et al., 2020b). FOXP3 can activate the Wnt/beta-catenin signaling pathway, thus, inducing epithelial–mesenchymal transition and promoting the progression of non-small cell lung cancer (Yang et al., 2017). In addition, the regulation of the expression of the NDUFB11 gene can regulate the programmed cell death process, so it promises to be a new target for cancer therapy (Panelli et al., 2013).

Furthermore, Kaplan–Meier survival curves in the train and entire set indicated that the patients with high risk score tend to have a worse prognosis. Besides, a survival ROC curve analysis demonstrated the predictive value of the OXPHOS-related prognostic signature. In addition, by conjoint univariate and multivariate analyses, the independence of the OXPHOS-related signature for prognosis prediction was verified. By combining the clinical features, we performed subgroup survival analysis in different risk groups and discovered that high risk was related to poor prognosis in all subgroups. Primarily, we observed that the risk score was highly correlated to tumor stage, tumor grade, histological type, and patient age. What is more, multi-ROC curve analyses and the evaluation of the nomogram provided evidence for the potential clinical application of the signature in prognosis prediction.

The results of GSEA indicated that the representative pathways in the low-risk group were mainly related to immunization. What is more, the immune score, stromal score, and ESTIMATE score of the low-risk group were higher, and the tumor purity was lower, which implied that patients with a low risk score appeared to be more immunogenic and might preferentially benefit from immunotherapy. To penetrate the immune mechanism of the signature, we compared TIICs between the two different risk groups and analyzed the relationship between the infiltrating immune cells and risk score. We found that plasma cells, CD8^+^ T cells, activated memory CD4^+^ T cells, and regulatory T cells (Tregs) were remarkably elevated in the low-risk group, while M0 macrophages, M2 macrophages, and activated mast cells were significantly elevated in the high-risk group. Immune cell profiles in the tumor microenvironment (TME) of UCEC tissues could be utilized to predict the survival of patients with UCEC (Li and Wan, 2020). Under the influence of environmental stimuli, tumor-associated macrophages (TAMs) can differentiate into two types of macrophage with opposite functions, in which M1 macrophages are protective immune cells that can remove pathogens and malignant cells. In contrast, M2 macrophages are dangerous factors, which can promote angiogenesis and the growth of tumor. High M2 macrophage infiltration in most tumor types is correlated with an adverse prognosis (Zhao et al., 2017; Wang et al., 2018), which is consistent with our results. A meta-analysis proved that UCEC patients with a high CD8^+^ T-cell density had a favorable prognosis (Guo et al., 2020). Increased expression of activated CD4^+^ memory T cells had been confirmed to be beneficial for prognosis in bladder cancer (Li et al., 2020), and the previous study of our team also provided evidence for the opinion (Liu et al., 2020c). The regulatory T cell (Treg) could suppress the antitumor immune response, thus, becoming a target of immunotherapy (Tanaka and Sakaguchi, 2017; Antomarchi et al., 2019), which is different from our results. However, another study showed that FOXP3 lymphocytic infiltration had no significant impact on the survival state of UCEC patients (Giatromanolaki et al., 2008). Our results suggested that CD8^+^ T cells, activated memory CD4^+^ T cells, regulatory T cells (Tregs), M0 macrophages, and M2 macrophages were crucial to the development of UCEC, which had the potential to be targeted for immunotherapies. The immune cell subpopulation enrichment analyses, as well as the ssGSEA-related functions or pathway analyses, showed that the scores of all the items except for aDCs and type I IFN response were higher in the low-risk group, which further proved that UCEC patients with low risk scores appeared to be more immunogenic.

Furthermore, patients with low risk scores had significantly higher scores of IPS, IPS-PD1-PD-L1-PD-L2, IPS-CTLA4, and IPS-PD1-PD-L1-PD-L2-CTLA4; thereby, they tend to respond more sensitively to immune checkpoint inhibitors. The Food and Drug Administration (FDA) has approved a series of monoclonal antibodies (mAbs) targeting PD-1, PD-L1, and CTLA-4 in several malignancy therapies (Arora et al., 2018). Besides, several clinical trials demonstrated the clinical value of anti-PD-1 antibody, pembrolizumab, which can inhibit tumor immune escaping in endometrial cancer. It has been approved as an alternative therapy for patients in the advanced stage (Musacchio et al., 2020). Also, the expression of CD27, CTLA4, ICOS, PD-L2, TIGIT, PD-1, LAG3, TIM-3, CD86, PD-L1, CD70, and CD270 were also remarkably higher in the low-risk group, which deserved to be explored as a new target for immunotherapy. TIGIT could enhance T-cell function by inhibiting PVRIG, so TIGIT-PVRIG pathways play a vital role in human cancers (Whelan et al., 2019). LAG-3 has been identified on various tumor cells, including UCEC, as a critical inducer for the malignancy progression (Friedman et al., 2020). There is also increasing evidence for TIM-3 as an emerging target for malignancy immunotherapy (Moore et al., 2019). The difference found in the comparison of the response to immune checkpoint blockade (ICB) therapy between different risk groups showed the same trend with the above findings. We also compared the risk score of the patients showing a different response to ICB, and found that the responders tended to possess a low risk score. These results demonstrated that the OXPHOS-related prognostic signature had the potential to be the classifier for a specific subset of UCEC patients, who would have a favorable effect on ICB therapy. Compared with UCEC patients in the advanced stage, patients with primary tumor tend to have more somatic loss of mismatch repair (MMR) protein expression; thus, the loss of MMR should be considered as guidance for immunotherapy (Ta et al., 2018). We compared the MSI status of different risk groups and found that the expression of dominant MMR proteins (MLH1, MSH2, MSH6, PMS2, and EPCAM) and mRNAsi in the low-risk group was downregulated, which illustrated that the patients with low risk score were burdened with high-MSI and showed fewer stemness features associated with oncogenic dedifferentiation. Haoya Xu et al. found that cell stemness can predict patient prognosis and constructed an mRNAsi-related prognostic signature in endometrial cancer (Xu et al., 2021). Accumulating evidence revealed that m^6^A mRNA methylation participates in the pathogenesis and progression of cancers through molecular mechanisms, such as inhibiting the antitumor response of CD8^+^ T cells (Chen et al., 2019; Sun et al., 2019). Patients with a low risk score had decreased gene expression of the m^6^A mRNA methylation, thus, showing a favorable response to immunotherapy.

To explore the cause of tumorigenesis, we compared the TMB of each group, and we discovered that the low-risk group had remarkably higher TMB, and the risk score was negatively correlated with TMB. It is reported that patients with high TMB had a more sensitive response to immune checkpoint blockade, such as anti-PD-1 agents (Fancello et al., 2019). A multicohort study demonstrated the value of tissue TMB (tTMB) as a predictive biomarker for response to pembrolizumab monotherapy (Marabelle et al., 2020). In our present study, the most frequent somatic mutations in the low-risk group were PTEN, ARID1A, PIK3CA, TTN, PIK3R1, CTCF, MUC16, ZFHX3, KMT2D, and MUC5B. PTEN functions as a tumor inhibitor by decreasing the activity of phosphoinositide 3-kinase (PI3K) (Bian et al., 2018), and its expression diminished with the progression of UCEC (Xi et al., 2019). It has been proven that ARID1A alterations indicated improved prognosis of tumor patients for compromising MMR and enhancing the infiltration of lymphocytes (Jiang et al., 2020). UCEC patients with high PIK3CA had shorter survival time because PIK3CA could change the tumor immune microenvironment by altering the fraction of tumor-associated neutrophils (Pan et al., 2019). Eighty-five percent of lung squamous cell carcinoma patients with TTN mutant type had missense variations and favorable overall survival (Cheng et al., 2019). In addition, patients in the high TMB group showed better overall survival than in the low TMB group, and the combined Kaplan–Meier analyses proved that risk score was a reliable predictive indicator independent of TMB. Our results were consistent with previous studies, indicating that UCEC patients with low risk score tend to have improved response to immunotherapy and favorable prognosis.

We compared the predicted effectiveness of chemotherapy drugs between different risk groups by using the GDSC dataset. Patients with UCEC in the low-risk group were more sensitive to temsirolimus, roscovitine, AZD6244, PD.0325901, RDEA119, PF.02341066, AKT. inhibitor.VIII, BMS.509744, vinblastine, bryostatin.1, metformin, AZ628, nutlin.3a, bortezomib, bicalutamide, AZD6482, cytarabine, rapamycin, camptothecin, mitomycin. C, S. Trityl.L.cysteine, tipifarnib, parthenolide, sorafenib, and methotrexate, which might be a potential helpful treatment option for UCEC.

These findings indicated that patients with low risk scores possessed higher IPS, higher TMB, and responded more sensitively to ICI and chemotherapy, which partly explained the reason for the improved prognosis of the low-risk patients.

However, there are still some deficiencies with our study. First of all, our data were based entirely on TCGA databases and lacked an external database for validation. Second, our molecular results lack the evidence of the experiments *in vitro* or *in vivo*. Third, these OXPHOS-related prognostic genes are supposed to be further examined by cell function assay for biological function and pathway analyses.

## 5 Conclusion

In conclusion, an OXPHOS-related and immune microenvironment prognostic signature classifying EC patients into different risk subsets was constructed in our study. This model could predict the prognosis of patients and help to select a specific subset of patients who might benefit from immunotherapy and chemotherapy, thus, improving the overall survival rate. These results may also contribute to the identification of novel immune biomarkers or target therapy in UCEC and give a new insight into the tumorigenesis and progression of UCEC.

## Data Availability

The datasets presented in this study can be found in online repositories. The names of the repository/repositories and accession number(s) can be found in the article/Supplementary Material.

## Supplementary Material

Supplementary Figure S1 Selected OXPHOS-related DEGs from uterine corpus endometrial carcinoma samples. Heatmap **(A)** and volcano plot **(B)** of OXPHOS-related DEGs between UCEC and normal endometrium. Red dots represented up-regulated OXPHOS-related DEGs, green dots represented down-regulated OXPHOS-related DEGs and black dots represented no OXPHOS-related DEGs. Functional annotation of the OXPHOS-related DEGs included Gene ontology analysis **(C)** and Kyoto Encyclopedia of Genes and Genomes pathway analysis **(D)**. **(E)** The PPI network indicated the interactions between OXPHOS-related DEGs.

Supplementary Figure S2 Construction of a prognostic model based on OXPHOS-related DEGs. **(A,B)** LASSO Cox regression analysis and multivariate Cox regression analysis. **(C)** Forest plots present the multivariate Cox proportional hazards regression analysis of the prognosis-associated OXPHOS-related DEGs.

Supplementary Figure S3
**(A–D)** The expression of the four prognostic OXPHOS-related DEGs in tumor (*N* = 511) and normal tissue (*N* = 23). **(E,F)** The relationship between ATP5IF1, FOXP3 and the overall survival of UCEC patients. **(G–J)** Expression levels of the four OXPHOS-related DEGs mRNA in clinical tissue samples.

Supplementary Figure S4 Stratified analysis. **(A–H)** The Kaplan-Meier survival analyses based on the prognostic signature stratified by tumor stage, tumor grade, histological type, patient age in the TCGA-UCEC cohort. **(I–L)** Relationship between the prognostic signature and tumor stage, tumor grade, histological type and age in the TCGA-UCEC cohort.

Supplementary Figure S5 The heatmap shows the OXPHOS-related prognostic genes expression and clinical characteristics of the TCGA-UCEC cohort.

Supplementary Figure S6 Variant classification, mutation type, SNV class, mutation load and top 10 mutated genes in UCEC between low **(A)** and high risk groups **(B)**. In the high risk group, *N* = 267; in the low risk group, *N* = 244.
