# Supplementary material for: Development of an Oxidative Phosphorylation-Related and Immune Microenvironment Prognostic Signature in Uterine Corpus Endometrial Carcinoma
Source: Front Cell Dev Biol. 2021 Nov 25;9:753004. doi: 10.3389/fcell.2021.753004 (PMC8655987; doi:10.3389/fcell.2021.753004)
Supplement: Supplementary file 1 [file Table1.DOCX]

Table 1. Clinical information of the included UCEC patients in TCGA.

| Covariates | Type | Total N (Percentage) | Train N (Percentage) |
| --- | --- | --- | --- |
| Age | ＜=60 | 199 (38.94%) | 109 (42.58%) |
|  | ＞60 | 312 (61.06%) | 147 (57.42%) |
| Histological type | Endometrial | 384 (75.15%) | 197 (76.95%) |
|  | Mixed and serous | 127 (24.85%) | 59 (23.05%) |
| Grade | G1 & G2 | 91 (17.81%) | 44 (17.19%) |
|  | G3 & G4 | 420 (82.19%) | 212 (82.81%) |
| Stage | Stage I & Stage II | 370 (72.41%) | 182 (71.09%) |
|  | Stage III Stage IV | 141 (27.59%) | 74 (28.91%) |
