# Supplementary figures and images for: Development of an Oxidative Phosphorylation-Related and Immune Microenvironment Prognostic Signature in Uterine Corpus Endometrial Carcinoma
Source: Front Cell Dev Biol. 2021 Nov 25;9:753004. doi: 10.3389/fcell.2021.753004 (PMC8655987; doi:10.3389/fcell.2021.753004)

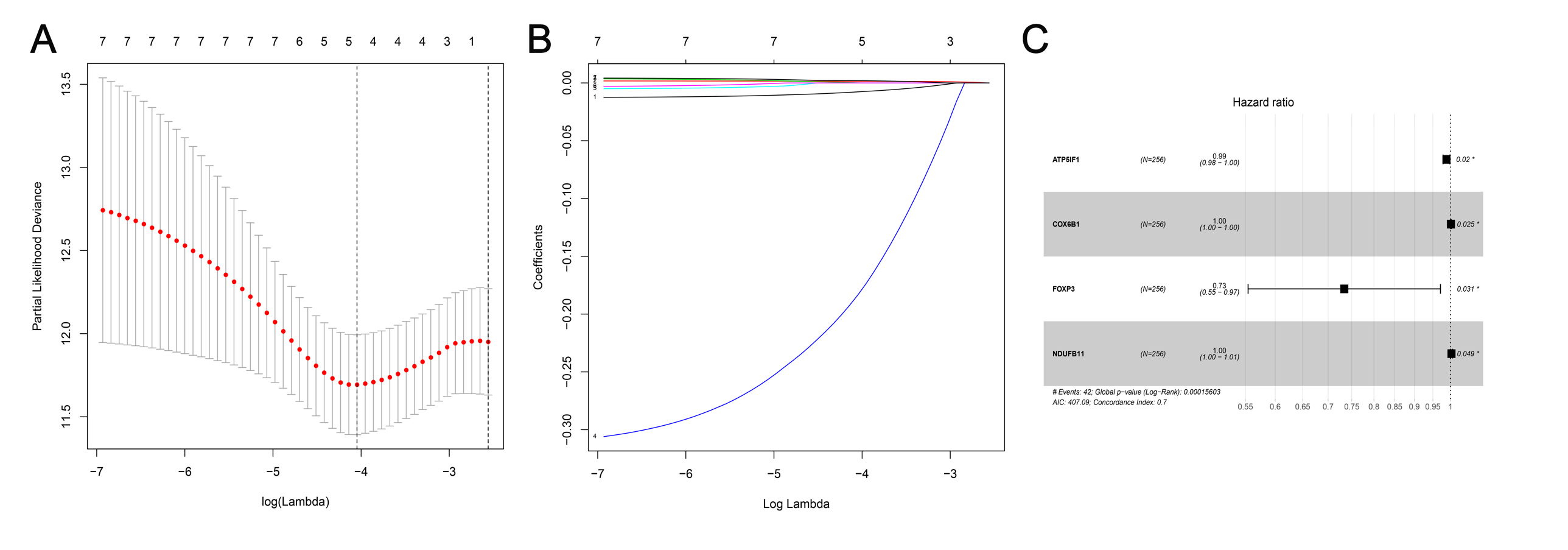

Supplement: Supplementary file 5 [file Image2.TIF]

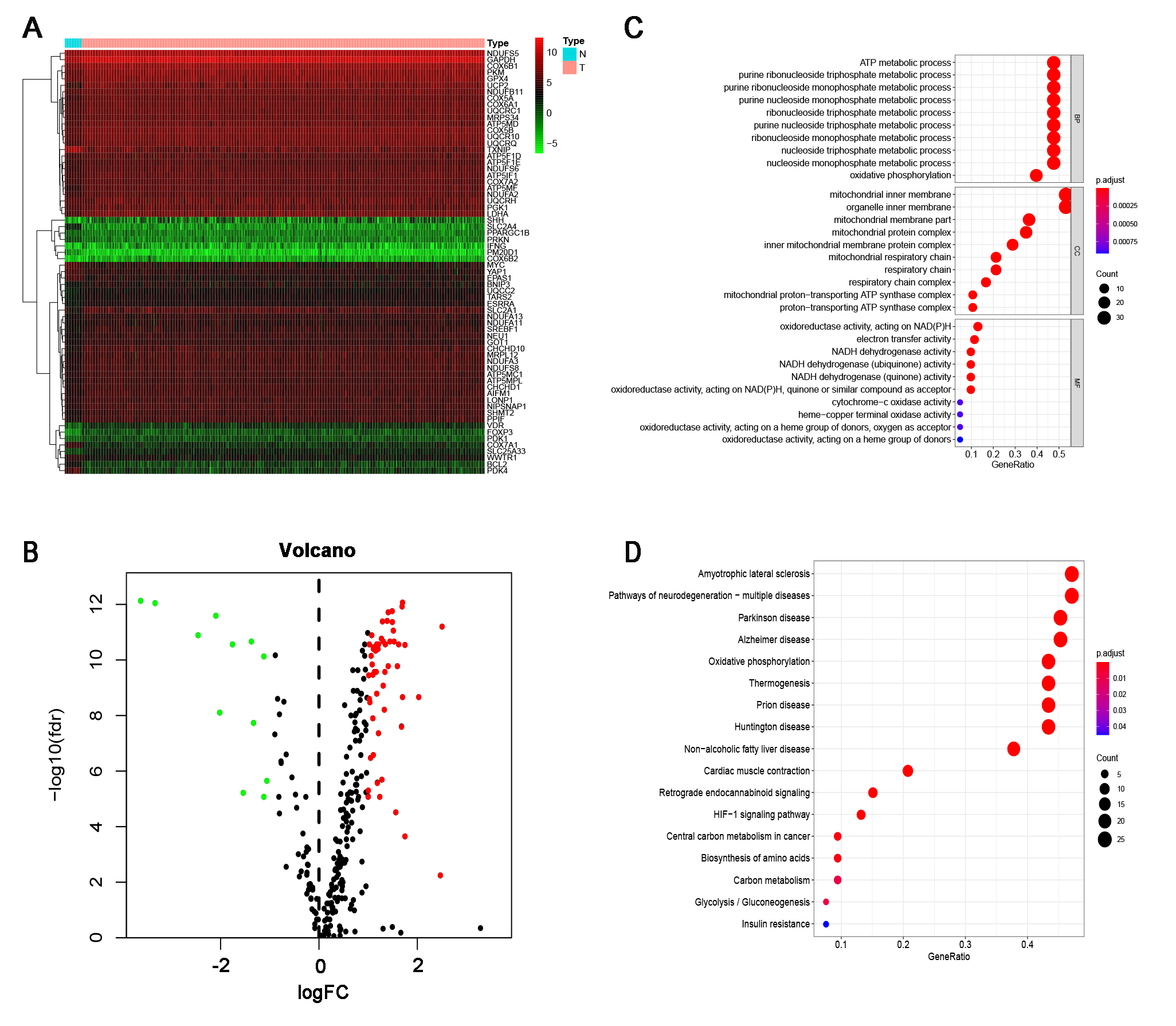

Supplement: Supplementary file 6 [file Image1.TIF]
