# Supplementary material for: Development of an Oxidative Phosphorylation-Related and Immune Microenvironment Prognostic Signature in Uterine Corpus Endometrial Carcinoma
Source: Front Cell Dev Biol. 2021 Nov 25;9:753004. doi: 10.3389/fcell.2021.753004 (PMC8655987; doi:10.3389/fcell.2021.753004)
Supplement: Supplementary file 7 [file Table2.DOCX]

Table S2 Primers’ information.

| Gene | Forward primer | Reverse primer |
| --- | --- | --- |
| GAPDH | 5' ACCACAGTCCATGCCATCAC 3' | 5' TCTAGACGGCAGG TCAGGTC 3' |
| ATP5IF1 | 5' CGATATTTCCGAGCACAGAGTAG 3' | 5' TGCTTATGGCGCTCAATTTCTTT 3' |
| COX6B1 | 5' CTACAAGACCGCCCCTTTTGA 3' | 5' GCAGAGGGACTGGTACACAC 3' |
| FOXP3 | 5' GTGGCCCGGATGTGAGAAG 3' | 5' GGAGCCCTTGTCGGATGATG 3' |
| NDUFB11 | 5' CGTCCGCTGGGAATCTAGC 3' | 5' ACGGGGTCCTTGTCATAACCA 3' |
