# Supplementary material for: Development of an Oxidative Phosphorylation-Related and Immune Microenvironment Prognostic Signature in Uterine Corpus Endometrial Carcinoma
Source: Front Cell Dev Biol. 2021 Nov 25;9:753004. doi: 10.3389/fcell.2021.753004 (PMC8655987; doi:10.3389/fcell.2021.753004)
Supplement: Supplementary file 8 [file Table3.DOCX]

Table S3

A total of 66 OXPHOS-related DEGs were screened out based on the TCGA-UCEC cohort.

| gene | conMean | treatMean | logFC | pValue | fdr |
| --- | --- | --- | --- | --- | --- |
| PGK1 | 39.0829587 | 78.3457446 | 1.003315228 | 2.03E-06 | 4.92E-06 |
| SHMT2 | 11.19270852 | 31.52500024 | 1.493937185 | 1.62E-13 | 4.25E-12 |
| BCL2 | 10.34140909 | 3.065134509 | -1.754410485 | 2.61E-12 | 2.72E-11 |
| COX5B | 55.1896413 | 140.4984012 | 1.3480843 | 2.29E-12 | 2.72E-11 |
| CHCHD10 | 12.85540387 | 41.68362587 | 1.697105841 | 4.90E-10 | 2.21E-09 |
| ATP5IF1 | 35.62555957 | 76.34659464 | 1.099651134 | 3.42E-09 | 1.23E-08 |
| PM20D1 | 0.400182311 | 0.098625447 | -2.020625561 | 2.05E-09 | 7.88E-09 |
| COX7A2 | 30.88929 | 62.68906632 | 1.021107132 | 2.73E-12 | 2.72E-11 |
| COX6B1 | 121.6144017 | 293.6282288 | 1.271676588 | 1.02E-12 | 1.67E-11 |
| MYC | 50.82886348 | 20.2273472 | -1.329340849 | 5.40E-09 | 1.86E-08 |
| COX7A1 | 25.08776552 | 4.581319354 | -2.453148942 | 7.27E-13 | 1.27E-11 |
| UQCR10 | 54.30530696 | 124.7267473 | 1.199605784 | 4.50E-12 | 3.93E-11 |
| PPARGC1B | 1.136250887 | 0.522529957 | -1.120695766 | 3.86E-06 | 8.42E-06 |
| UCP2 | 56.95208265 | 134.4827602 | 1.239600736 | 3.96E-06 | 8.48E-06 |
| PPIF | 14.11365735 | 35.47804705 | 1.329834707 | 1.55E-09 | 6.15E-09 |
| COX6B2 | 0.075777699 | 0.254511168 | 1.747883726 | 0.00012606 | 0.000224678 |
| NEU1 | 9.902071348 | 26.20675543 | 1.4041365 | 4.46E-14 | 1.95E-12 |
| LONP1 | 16.63286304 | 33.80506441 | 1.023202872 | 5.67E-10 | 2.46E-09 |
| NIPSNAP1 | 16.38050591 | 36.91079572 | 1.172062924 | 3.38E-10 | 1.64E-09 |
| YAP1 | 39.49964652 | 18.14475588 | -1.122287096 | 1.03E-11 | 7.27E-11 |
| UQCRC1 | 44.14145565 | 92.15357853 | 1.061905984 | 9.65E-12 | 7.08E-11 |
| NDUFA3 | 15.17729278 | 40.2833486 | 1.408269138 | 2.57E-11 | 1.68E-10 |
| MRPL12 | 12.80365396 | 43.12701756 | 1.752036359 | 3.04E-12 | 2.85E-11 |
| ATP5MPL | 12.63183209 | 30.95036883 | 1.292892703 | 1.41E-13 | 4.09E-12 |
| AIFM1 | 16.01698117 | 37.05608634 | 1.210108261 | 1.39E-08 | 4.40E-08 |
| ATP5F1D | 33.29778565 | 66.64455877 | 1.001060851 | 3.72E-06 | 8.25E-06 |
| FOXP3 | 0.307465164 | 1.737739112 | 2.498716639 | 2.60E-13 | 6.18E-12 |
| NDUFS5 | 226.9379174 | 523.9129116 | 1.207029339 | 2.73E-12 | 2.72E-11 |
| SLC25A33 | 1.858998339 | 5.359913929 | 1.527684352 | 1.68E-12 | 2.20E-11 |
| SLC2A1 | 13.69791652 | 55.92820479 | 2.02961955 | 4.90E-10 | 2.21E-09 |
| VDR | 1.269172057 | 4.072931862 | 1.682180016 | 7.45E-09 | 2.50E-08 |
| NDUFA13 | 9.756861 | 24.18379007 | 1.30955138 | 1.66E-10 | 8.51E-10 |
| ATP5MC1 | 16.91807648 | 34.6650269 | 1.034915331 | 8.10E-10 | 3.32E-09 |
| COX5A | 31.75437304 | 83.14073418 | 1.388600202 | 1.17E-13 | 3.83E-12 |
| ATP5F1E | 26.44127826 | 53.31699862 | 1.011803646 | 6.53E-11 | 3.49E-10 |
| ATP5MF | 20.4961213 | 66.5848198 | 1.699842386 | 6.62E-15 | 8.67E-13 |
| TARS2 | 5.830574391 | 12.59354426 | 1.110974444 | 4.71E-12 | 3.98E-11 |
| NDUFS6 | 27.08759478 | 66.96550665 | 1.305785871 | 1.54E-12 | 2.20E-11 |
| PDK4 | 47.50277465 | 4.723053631 | -3.330219961 | 1.03E-14 | 9.01E-13 |
| SHH | 0.653285119 | 1.935336005 | 1.566799381 | 1.58E-05 | 3.09E-05 |
| NDUFA2 | 30.15412609 | 64.42918314 | 1.095358879 | 6.10E-11 | 3.33E-10 |
| CHCHD1 | 15.65268739 | 33.59660598 | 1.101905122 | 3.79E-12 | 3.42E-11 |
| TXNIP | 404.9900261 | 94.71262711 | -2.096257694 | 6.66E-14 | 2.49E-12 |
| UQCRQ | 40.57098435 | 113.8834065 | 1.489037341 | 3.38E-14 | 1.77E-12 |
| UQCRH | 48.67517783 | 100.8218752 | 1.050550539 | 1.18E-07 | 3.33E-07 |
| IFNG | 0.097864676 | 0.54116307 | 2.467203274 | 0.004014018 | 0.005746846 |
| GAPDH | 425.991887 | 1378.453866 | 1.694153125 | 1.85E-14 | 1.21E-12 |
| NDUFS8 | 14.85616709 | 40.38413901 | 1.442726835 | 1.63E-12 | 2.20E-11 |
| PDK1 | 0.828163013 | 1.886037368 | 1.187371584 | 1.05E-06 | 2.63E-06 |
| SREBF1 | 8.743571174 | 26.42133443 | 1.595408782 | 2.44E-11 | 1.64E-10 |
| PRKN | 1.405955117 | 0.542649503 | -1.373457971 | 1.47E-12 | 2.20E-11 |
| MRPS34 | 38.62600609 | 86.40859113 | 1.161602247 | 4.79E-11 | 2.67E-10 |
| ATP5MD | 43.83617435 | 95.69369216 | 1.126301932 | 4.55E-11 | 2.65E-10 |
| PKM | 75.1337787 | 214.0208187 | 1.510217574 | 3.94E-13 | 8.61E-12 |
| UQCC2 | 5.017662087 | 11.07766922 | 1.142567144 | 4.79E-11 | 2.67E-10 |
| NDUFB11 | 48.23905 | 107.0716718 | 1.150303433 | 5.84E-12 | 4.68E-11 |
| EPAS1 | 45.88811996 | 15.74626822 | -1.543110742 | 2.59E-06 | 6.01E-06 |
| SLC2A4 | 5.273490909 | 0.427463376 | -3.624885579 | 2.78E-15 | 7.28E-13 |
| LDHA | 39.38077696 | 121.3022478 | 1.623042805 | 2.23E-12 | 2.72E-11 |
| ESRRA | 6.486955435 | 13.73300317 | 1.082033719 | 2.12E-11 | 1.46E-10 |
| NDUFA11 | 9.579160609 | 20.62057519 | 1.106113428 | 8.92E-08 | 2.60E-07 |
| COX6A1 | 40.58020174 | 91.68536451 | 1.175915421 | 2.68E-12 | 2.72E-11 |
| WWTR1 | 18.36864374 | 8.810003221 | -1.060030656 | 8.84E-07 | 2.25E-06 |
| GPX4 | 77.67360739 | 196.8890978 | 1.341886853 | 4.40E-11 | 2.62E-10 |
| GOT1 | 10.7140973 | 22.56225589 | 1.074401019 | 6.94E-13 | 1.27E-11 |
| BNIP3 | 6.730015217 | 16.30910236 | 1.276995708 | 7.86E-07 | 2.02E-06 |
