# Supplementary material for: Development of an Oxidative Phosphorylation-Related and Immune Microenvironment Prognostic Signature in Uterine Corpus Endometrial Carcinoma
Source: Front Cell Dev Biol. 2021 Nov 25;9:753004. doi: 10.3389/fcell.2021.753004 (PMC8655987; doi:10.3389/fcell.2021.753004)
Supplement: Supplementary file 9 [file Table4.DOCX]

Table S4. A total of 7 OXPHOS-related prognostic DEGs were further filtrated in the patients with UCEC

| id | HR | HR.95L | HR.95H | P value |
| --- | --- | --- | --- | --- |
| ATP5IF1 | 0.990 | 0.981 | 1.000 | 0.050 |
| COX6B1 | 1.002 | 1.001 | 1.003 | 0.000 |
| MRPL12 | 1.006 | 1.000 | 1.012 | 0.047 |
| FOXP3 | 0.725 | 0.547 | 0.961 | 0.025 |
| NDUFA13 | 1.008 | 1.000 | 1.016 | 0.046 |
| ATP5F1E | 1.006 | 1.000 | 1.013 | 0.049 |
| NDUFB11 | 1.003 | 1.001 | 1.006 | 0.006 |
